# Postpartum well-being in hemophilia carriers and women with von Willebrand disease: insights from patient-reported outcome measures

**DOI:** 10.1016/j.rpth.2026.103418

**Published:** 2026-03-24

**Authors:** Anne de Vaan, Elke A. Doeff, Jeroen Eikenboom, Marieke J.H.A. Kruip, Marieke C. Punt, Michiel Coppens, Laurens Nieuwenhuizen, Saskia E.M. Schols, Anja B.U. Mäkelburg, Floor C.J.I. Heubel-Moenen, Hans J. Duvekot, Marjolein Peters, Annemieke Middeldorp, Kitty W.M. Bloemenkamp, Roger E.G. Schutgens, Karin P.M. van Galen

**Affiliations:** 1 Division of Internal Medicine and Dermatology, Center for Benign Haematology, Thrombosis and Haemostasis, van Creveldkliniek, University Medical Center Utrecht, Utrecht University, Utrecht, Netherlands; 2 Department of Internal Medicine, Division of Thrombosis and Hemostasis, Leiden University Medical Center, Leiden, Netherlands; 3 Department of Hematology and department of Quality and Patient Care, Erasmus MC, Erasmus University Medical Center Rotterdam, Rotterdam, Netherlands; 4 Department of Vascular Medicine, Academic Medical Center, University of Amsterdam, Amsterdam, Netherlands; 5 Department of Haematology, Maxima Medisch Centrum, Veldhoven, Netherlands; 6 Department of Hematology, Radboud University Medical Center and Hemophilia Treatment Center Nijmegen-Eindhoven-Maastricht, Nijmegen, Netherlands; 7 Department of Hematology, University Medical Center Groningen, Groningen, Netherlands; 8 Department of Hematology-Internal Medicine, Maastricht University Medical Center+ and Hemophilia Treatment Center Nijmegen-Eindhoven-Maastricht and CARIM, Maastricht University, Maastricht, Netherlands; 9 Department of Obstetrics, Erasmus MC, Erasmus University Medical Center, Rotterdam, Netherlands; 10 Department of Pediatric Hematology, Emma Children’s Hospital, University Medical Centers, Location AMC, Amsterdam, Netherlands; 11 Department of Obstetrics, Leiden University Medical Center, Leiden, Netherlands; 12 Department of Obstetrics, WKZ Birth Centre, Division Woman and Baby, University Medical Center Utrecht, Utrecht, Netherlands

**Keywords:** hemophilia, quality of life, patient-reported outcome measures, postpartum period, von Willebrand disease

## Abstract

**Background:**

Hemophilia carriers (HCs) and women with von Willebrand disease (VWD) receive specialized obstetric care because of a higher chance for postpartum bleeding and potential bleeding in the neonates. It is unknown what their postpartum quality of life (QoL), childbirth satisfaction, and experience are and how this differs from the general population.

**Objectives:**

This study assessed QoL, childbirth satisfaction and experience in HCs and women with VWD at week 1 and 6 postpartum. These outcomes are compared with those from retrospective studies of the general population.

**Methods:**

Participants completed 3 patient-reported outcome measures postpartum: the Short Form-36 at week 1 and 6 measuring QoL, the Mackey Childbirth Satisfaction Rate Scale at week 1 for childbirth satisfaction, and the Labor and Delivery Index at week 6 for childbirth experience. Descriptive statistics were used.

**Results:**

In total, 85 HCs and 81 women with VWD completed ≥1 questionnaire. Pain and physical functioning improved over time (both moderate to fairly well; *P* < .001). Six weeks postpartum, QoL was lower in both groups than those in the general population. Over 88% of both cohorts reported “at least satisfied” on the Mackey Childbirth Satisfaction Rate Scale, significantly higher than the general population (>61%; *P* < .001). Mean Labor and Delivery Index scores (1.3-1.9 points) indicated an adequate childbirth experience. HCs reported more child-related worries than the general population (37.3% vs 72.2%; *P* < .001).

**Conclusion:**

HCs and women with VWD recover less between week 1 and 6 postpartum than the general population. HCs report more worries about their child during childbirth than women with VWD and the general population.

## Essentials

• Patient-reported outcome measures (PROMs) in postpartum women with a bleeding disorder are unknown.
• Three PROMs were completed postpartum: Short Form-36, MCSRS, and the LADY-X.
• Quality of life improved between week 1 and 6 postpartum. Childbirth satisfaction was high.
• Hemophilia carriers had more worries about the child than the general population.

## 1 Introduction

Hemophilia carriers (HCs) and women with von Willebrand disease (VWD) have impaired hemostasis due to lower factor (F)VIII, FIX, or von Willebrand factor (VWF) levels. This can result in excessive and prolonged bleeding during hemostatic events, such as childbirth. To minimize the risk for severe postpartum hemorrhage (PPH; ≥1000 mL), these women receive prophylactic desmopressin, FVIII, FIX, or VWF concentrate during childbirth if their third trimester activity levels are <80 IU/dL. Those who receive supplementation require factor levels of >50 IU/dL for at least 3 days after vaginal birth and 5 days after a cesarean section (CS). Furthermore, prophylactic tranexamic acid is prescribed in all deliveries when flooding persists (in general, if the need to change pads exceeds every 3 hours) [1,2]. To minimize the bleeding risk in the potentially affected or proven affected neonates, an alternative birth plan is designed, which, for example, recommends to withhold from assisted vaginal deliveries [[1], [2], [3]].

Excessive bleeding may cause severe physical health problems, such as shock and anemia [4]. Furthermore, the increased risk for prolonged hospitalizations and emergency interventions can also have long-term consequences such as posttraumatic stress disorder (PTSD) [5]. All these factors may potentially impact postpartum quality of life (QoL) [6,7]. A positive childbirth experience can improve the mothers’ well-being, while negative experiences can have a detrimental impact on her self-esteem and overall mental well-being [8], which can extend beyond childbirth [9]. Childbirth satisfaction can plays an important role in the relationship between mother and child. It impacts women’s self-esteem [10]. Unsatisfactory childbirths can have a negatively influence, leading to possible postpartum depression or PTSD. It can also result in future abortions, preference for future CSs, or hesitation about engaging in intercourse [11].

It is currently unknown how hematologic and obstetric management in HCs and women with VWD influences their postpartum QoL, childbirth satisfaction, and childbirth experience. This study aims to describe patient-reported outcome measures (PROMs) in HCs and women with VWD in the postpartum period. Additionally, we aim to analyze the difference in QoL childbirth experience, and childbirth satisfaction between HCs and women with VWD compared to the general Dutch population.

## 2 Methods

This study is part of the PRegnancy and Inherited bleeding DisordErS (PRIDES) study, which prospectively collected obstetric and hematologic data from HCs and women with VWD delivering under the updated 2018 Dutch guideline. Since then, we offer enhanced hemostatic management when third trimester VWF/FVIII/FIX levels are <80 IU/dL instead of <50 IU/dL. Peak target levels at delivery are now ≥150 IU/dL instead of ≥100 IU/dL, with trough levels of ≥50 IU/dL. The clinical outcomes are intended to be reported separately [1,2]. The PRIDES study was deemed outside the scope of the Medical Research Involving Human Subjects Act (WMO), by the UMCU Medical Ethics Research Committee (reference 17-792, the Netherlands National Trial Register NL6770).

HCs, women with hemophilia, and women with VWD (≥18 years) with pregnancies beyond 10 weeks who visited 1 of 6 Dutch hemophilia treatment centers were invited to participate. To increase the sample size, HCs and women with hemophilia were pooled together in the analysis and are from now on referred to with the overarching term HCs. After informed consent, they completed the PROMs at week 1 postpartum (Short Form [SF]-36 and the Mackey Childbirth Satisfaction Rate Scale [MCSRS]) and week 6 (SF-36 and the Labor and Delivery Index [LADY-X]). Outcomes of the PROMs were compared with historical Dutch cohorts [[12], [13], [14]].

### 2.1 Statistical analysis

Questionnaires were included in analysis if >90% of the questionnaire was completed. Missing PROM data were imputed per domain using the average score if >50% of the items were completed; otherwise, cases were excluded from that domain analysis. Continuous variables were assessed via boxplots and Q-Q plots and reported as mean and SD or median and IQR. Categorical data are reported as frequencies and percentages. Analysis were conducted in SPSS Statistics 29.0 (IBM Corp) and RStudio 3.4 (R Foundation for Statistical Computing) [15,16].

### 2.2 Short Form-36

The SF-36 is a 36-item questionnaire evaluating QoL across 8 health domains: physical functioning, role limitations—physical health, role limitations—emotional, vitality, emotional well-being, social functioning, pain, and general health. Each domain has a various number of items, with each item being scored on a scale of 0 to 100. Outcomes are transformed according to the guidelines from the SF-36 developers. After transformation, higher domain scores indicate better QoL on that item [17]. Scores of <40 are classified as bad, 40 to 59 as moderate, 60 to 79 as fairly well, and 80 to 100 as extremely well [17].

Domain scores were assessed at week 1 and week 6 postpartum; mean differences were analyzed by a Student’s *t*-test. The mean difference in domain scores from the participants that completed both week 1 and week 6 are analyzed by a paired *T*-test.

Outcomes were compared with SF-36 data from a historical Dutch cohort of 141 women with 141 singleton deliveries between 2003 and 2004 by a 1-sample *T*-test [13]. This Dutch cohort contained 71 vaginal deliveries and 70 CSs from 3 hospitals. The mean age was between 30.5 (for both vaginal deliveries and elective CSs, *n* = 107) and 32.4 years (emergency CS, *n* = 34).

### 2.3 Mackey Childbirth Satisfaction Rate Scale

The MCSRS assesses the satisfaction with the interaction between mother and the caregivers and their partner. It is a 37-item questionnaire with 6 domains: self, partner, baby, nurse, physician, and general, and uses a 5-point Likert scale (0, very unsatisfied; 5, very satisfied) [18,19]. Overall, a score of <3.0 is categorized as dissatisfied, 3.0 to 3.9 as neutral, and ≥4.0 as satisfied. Total domain scores were calculated by multiplying the number of items by 5, with higher scores indicating greater satisfaction. The mean score per domain calculated by dividing the total domain score by 5.

The outcomes of the MSCRS were compared with those of a 2005 Dutch-Belgian cohort of 611 women with 611 deliveries [12]. The mean age was 31 years (range, 19-44 years), and 45.8% (*n* = 276/611) were primipara. This study reported the frequency of the responses satisfied and very satisfied together. Therefore, the outcomes of the MSCRS in our cohort were converted to a similar variable. Comparisons were made using a 1-sample test for proportion.

### 2.4 Labor and Delivery Index

The LADY-X is a subjective evaluation of labor and delivery by domains that reflect key aspects of the mother’s overall experience of labor and childbirth. It contains 7 domains: availability, information, needs, emotional support, safety, concerns, and first contact. Scoring is done by using a 3-point Likert scale (0, inadequate; 1, adequate; and 2, very well) [20].

LADY-X results were compared with those of a Dutch cohort of 295 deliveries from 295 women between June and October 2013 [14]. The mean age was 31.7 years (SD, 3.9 years), and 50% (*n* = 146) were primiparous. Vaginal deliveries occurred in 75% of the births (*n* = 222). The study reported the frequency of the responses very well and adequate together. Our results were therefore also analyzed by pooling the frequencies of very well and adequate together. Comparisons were made using a 1-sample test for proportions.

## 3 Results

**Figure 1:**
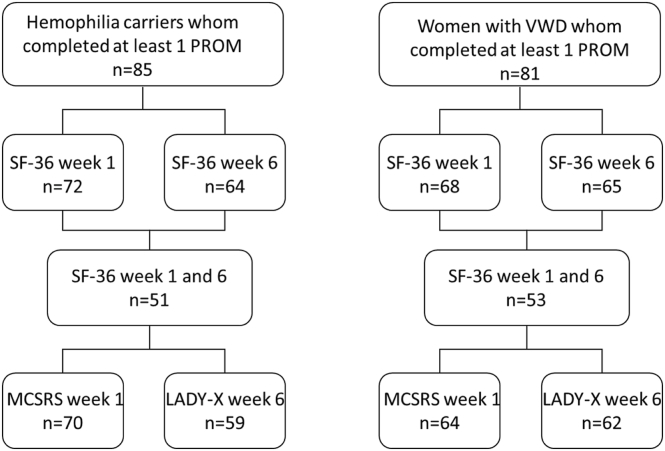
Flowchart of the number of participants who completed each patient-reported outcome measure (PROM). LADY-X, Labor and Delivery Index; MSCRS, Mackey Childbirth Satisfaction Rate Scale; n, number; SF, Short Form; VWD, von Willebrand disease.

Between September 2018 and March 2024, 170 HCs and 160 women with VWD enrolled in the PRIDES study. Overall, 81 women did not start the questionnaires at week 1 or completed <90% (SF-36 + MCSRS, 24.5%; *n* = 81/330); 95 women did not start the questionnaires or completed <90% at week 6 (SF-36 + LADY-X, 28.8%; *n* = 95/330). In total, 85 HCs (85.9% hemophilia A; *n* = 73/85) and 81 women with VWD (76.5% VWD type 1; *n* = 62/81) completed at least 1 of the PROMs. Figure 1 gives an overview of the completion rate of each PROM. The median gravidity was 2 (IQR, 1-2) in HCs and 2 (IQR, 1-3) in women with VWD. The median parity in HCs was 0 (IQR, 0-1) and 1 (IQR, 0-1) in women with VWD. Vaginal deliveries were the most prevalent (81%, *n* = 69/85, in carriers; 79%, *n* = 64/81, in women with VWD). Thrombosis or postpartum infections were not reported. Baseline parameters and obstetric outcomes are presented in Table 1.

**Table 1:** Baseline and delivery characteristics.

Variable	Hemophilia carriers^a^ (*n* = 85)	Women with VWD^a^ (*n* = 81)
Age (y)	32 (29-35)	33 (30-35)
Disease subtype		
Hemophilia A	73 (85.9)	NA
Hemophilia B	12 (14.1)	NA
VWD type 1	NA	62 (76.5)
VWD type 2A	NA	7 (8.6)
VWD type 2B	NA	6 (7.4)
VWD type 2M	NA	5 (6.2)
VWD type 2N	NA	1 (1.2)
VWD type 3	NA	0 (0)
Baseline FVIII levels < 40 IU/dL	9 (10.6)	NA
Baseline FIX levels < 40 IU/dL^b^	5 (41.7)	NA
Gestational age (wk)	40 (39-41)	40 (39-40)
Gravidity	2 (1-2)	2 (1-3)
Parity	0 (0-1)	1 (0-1)
Primiparity	45 (52.9)	34 (42.0)
History of PPH	9 (10.6)	22 (27.2)
Delivery mode		
Vaginal delivery	69 (81.2)	64 (79.0)
Elective cesarean section	7 (8.2)	10 (12.3)
Emergency cesarean section	9 (10.6)	7 (8.6)
Blood loss postpartum (mL)	382.5 (200.0-732.5)	350.0 (250.0-730.0)
Blood loss categories		
Severe PPH (≥1000 mL)	15 (17.6)	13 (16.0)
Mild PPH (500-999 mL)	17 (20.0)	16 (19.8)
Normal (<500 mL)	53 (62.4)	52 (64.2)
Episiotomy	15 (21.7)	8 (9.9)
Perineal laceration	34 (40.0)	22 (27.2)
Uterine atony	6 (7.1)	7 (8.6)
Placental retention	8 (9.4)	3 (3.7)
Neonatal bleeding disorder diagnosis		
Yes	15 (17.6)	14 (17.3)
No	25 (29.4)	10 (12.3)
Not yet known	45 (53.0)	57 (70.4)

Baseline and obstetric characteristics of the participants who completed at least 1 PROM. Values are *n* (%) or media (IQR).

NA, not available; PPH postpartum hemorrhage; VWD von Willebrand disease.

a Ethnicity was not collected since the Netherlands has a diverse ethnic/cultural population and the includied hemophilia treatment centers are from all regions in the Netherlands.

b Baseline FIX levels: 22.0 (*n* = 2), 31.0 (*n* = 1), 34.0 (*n* = 1), and 39.0 (*n* = 1).

Subjects who completed the PROMs had median 365 mL postpartum blood loss (IQR, 209-740 mL), compared with a median of 300 mL (IQR, 200-500 mL) in the PRIDES subjects who did not complete 1 of the PROMs (*P* = .13).

### 3.1 Short Form-36

The SF-36 was completed by 84.7% (*n* = 72/85) of the HCs at week 1 and by 75.3% (*n* = 64/85) at week 6 postpartum. Both week 1 and week 6 were completed by 60.0% (*n* = 51/85). Of the women with VWD, week 1 was completed by 84.0% (*n* = 68/81) and week 6 by 80.2% (*n* = 65/81). Fifty-three women with VWD completed both time points. A sensitivity analysis of the participants who completed both week 1 and week 6 showed no differences compared with participants with full data sets at week 1 and week 6 data (available on request). Therefore, we decided to analyze all participants who completed the SF-36 at 1 or 2 time points.

#### 3.1.1 Hemophilia carriers

**Figure 2:**
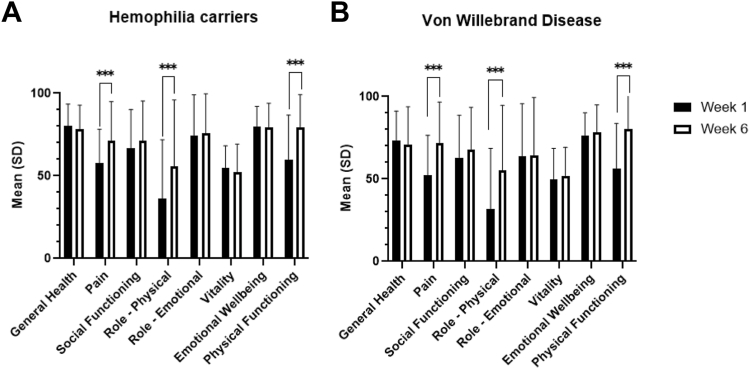
Data were normally distributed. Mean (SD) Short Form-36 outcomes in between week 1 and 6 postpartum in the cohorts. (A) Hemophilia carriers; (B) women with von Willebrand disease. Significance levels: ∗*P* < .05; ∗∗*P* < .01; ∗∗∗*P* < .001.

General health was reported as extremely well at week 1 (mean score, 80.9; SD, 14.1). At week 6, general health decreased toward fairly well, although it was reaching the upper boundary of that category (mean score, 78.3; SD, 14.5). Overall, the participants had a significant improvement of pain at week 6, with the reported scores changing from moderate to fairly well (mean score, 55.8; SD, 20.2, vs mean, 71.1; SD, 23.8; *P* < .001). The physical- and social functioning domains significantly improved at week 6 (moderate to fairly well [*P* < .001] and fairly well to fairly well [*P* = .04], respectively). Similarly, an increase in the role limitations (physical) domain was observed, albeit remaining moderate (mean score, 40.0; SD, 22.0, vs mean, 55.9; SD, 40.0; *P* < .001). The other domains remained stable: role limitations—emotions, fairly well; vitality, moderate; and emotional well-being, extremely well. Figure 2A displays the comparisons between week 1 and week 6 postpartum for all domains.

#### 3.1.2 Women with VWD

Overall, participants reported their general health at both week 1 and week 6 as very well. Pain improved significantly over time, increasing from 52.2 (SD, 24.2, moderate) to 71.9 (SD, 24.7, fairly well; *P* < .001). There was a significant increase in reported QoL on the physical functioning domain, improving from 56.4 (SD, 27.2, moderate) to 80.0 (SD, 20.8, extremely well; *P* < .001). Additionally, less role limitations due to physical restrictions were reported, with a significant increase from 31.5 (SD, 37.0, bad) to 55.3 (SD, 39.3, moderate; *P* < .001). All other domains remained stable between week 1 and week 6: social functioning, fairly well; role limitations—emotions, fairly well; vitality, moderate; and emotional well-being, fairly well (Figure 2B).

#### 3.1.3 Comparisons with the general population

**Figure 3:**
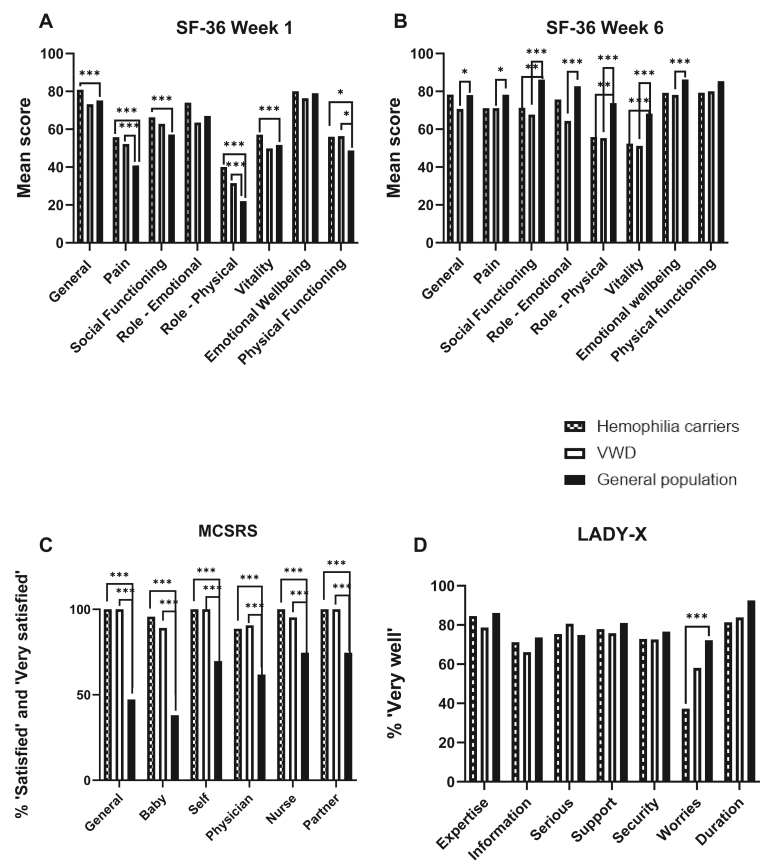
Comparisons of hemophilia carriers and women with von Willebrand disease between the general Dutch/Belgian populations. (A) SF-36 at week 1 postpartum. (B) Short Form-36 at week 6 postpartum. (C) MSCRS at week 1 postpartum. (D) LADY-X at week 6 postpartum. Significance levels: ∗ <0.05, ∗∗ <0.01, ∗∗∗ <0.001. LADY-X, Labor and Delivery Index; MSCRS, Mackey Childbirth Satisfaction Rate Scale; SF, Short Form; VWD, von Willebrand disease.

The mean scores of the SF-36 domains from the participants were compared with the SF-36 outcomes of 141 deliveries from the general Dutch population [13]. At week 1, the HCs reported higher QoL on all domains except the emotional well-being and role limitations (emotions) domains. These domains remained stable (Figure 3A, Supplementary Table 1). At 6 weeks postpartum, HCs had significantly lower scores on the social functioning, role limitations (emotions and physical), and energy domains (*P* < .05) (Figure 3B, Supplementary Table 1).

Women with VWD reported significantly less pain (mean scores, 52.2 vs 40.9; *P* < .001) and fewer role limitations due to physical health (mean scores, 31.5 vs 22.2; *P* = .03) compared with the general population (Supplementary Table 2) at week 1 postpartum. At week 6 postpartum, they reported significantly lower scores on all domains, except physical functioning. Figure 3A, B depicts the comparisons with the general population at both time points.

### 3.2 Mackey Childbirth Satisfaction Rate Scale

The total median MSCRS score for HCs was 148.4 (SD 18.2). On all domains, a satisfied childbirth satisfaction was scored, with the exception of general and self (neutral). Women with VWD had a total MCSRS of 148.1 (SD, 20.2) in the cohort, corresponding with an overall satisfactory child birth. All participants were satisfied with their childbirth and the interaction between them and the caregivers or their partner. The participants were most satisfied with their partner (Table 2).

**Table 2:** Outcomes of the Mackey Childbirth Satisfaction Rate Scale in postpartum hemophilia carriers and women with VWD.

Domain (maximum score)	Hemophilia carriers (*n* = 70)	Women with VWD (*n* = 64)
Total (185.0)	148.4 (18.2)	148.1 (20.2)
General (35.0)	22.7 (3.4)	22.9 (4.3)
Baby (15.0)	13.4 (2.1)	13.5 (2.5)
Self (45.0)	31.8 (6.5)	32.1 (6.7)
Physician (40.0)	34.6 (6.2)	34.1 (6.8)
Nurse (40.0)	36.2 (5.0)	35.9 (5.0)
Partner (10.0)	9.6 (0.9)	9.8 (0.6)

Data were normally distributed. Mean (SD) Mackey Childbirth Satisfaction Rate Scale scores for hemophilia carriers and women with VWD.

VWD, von Willebrand disease.

#### 3.2.1 Comparisons with the general population

When converting the outcomes of the MCSRS results from HCs to the percentage very satisfied (=5) and satisfied (=4) revealed that >88% of the participants were satisfied or very satisfied with their childbirth. In women with VWD, this was >89%. Both where significantly higher on all domains in comparison with the general population were 38.0% was at least satisfied with the interaction between themselves and their baby, and >61% at the other domains (Figure 3C, Supplementary Tables 3 and 4).

### 3.3 Labor and Delivery Index (LADY-X)

The mean scores on the LADY-X domains from HCs ranged between 1.3 and 1.8, corresponding with an overall adequate childbirth experience (Table 3). Most HCs experienced their childbirth as very well, and only a few carriers reported their childbirth as inadequate (Supplementary Table 3). This was mostly due to worries about their child (percentage inadequate: 11.1%; *n* = 6/54) (Table 3).

**Table 3:** Outcomes of the LADY-X in postpartum hemophilia carriers and women with VWD.

LADY-X domain	Hemophilia carriers (*n* = 59)		Women with VWD (*n* = 62)	
	Mean (SD)	Very well, *n* (%)	Mean (SD)	Very well, *n* (%)
Presence of expert health care	1.8 (0.4)	49 (83.1)	1.8 (0.5)	48 (77.4)
Information received	1.7 (0.5)	42 (71.2)	1.6 (0.5)	42 (67.8)
Taking wishes seriously	1.7 (0.5)	43 (72.9)	1.8 (0.5)	52 (83.8)
Emotional support	1.8 (0.5)	46 (80.0)	1.8 (0.4)	49 (79.0)
Feeling of security	1.7 (0.5)	43 (72.9)	1.7 (0.5)	48 (77.4)
Worries about child	1.3 (0.7)	26 (44.1)	1.6 (0.7)	42 (67.8)
Time until first contact with child	1.8 (0.5)	48 (81.4)	1.9 (0.3)	58 (93.2)

Mean (SD) LADY-X scores from hemophilia carriers and women with VWD. Mean scores per domain are given, together with the combined frequency of very well and adequate per domain. A score of 0 indicates inadequate; 1, adequate; and 2, very well.

LADY-X, Labor and Delivery Index; VWD, von Willebrand disease.

Mean scores from women with VWD were between 1.6 and 1.9, indicating adequate childbirth experience. Overall, >67% of the participants rated their childbirth experience as very well on all domains (Table 3).

#### 3.3.1 Neonatal bleeding disorder diagnosis

In total, 22% (*n* = 13/59) of the HCs who completed the LADY-X had a child with a confirmed bleeding disorder diagnosis. In 18.6% of the neonates, diagnostics revealed no disease. In 59.3%, it was not known if the neonate was affected. Of the 6 carriers who reported worries about their child, 1 knew her child had a bleeding disorder at birth, 1 had confirmation that her child did not have the disease, and 4 did not perform diagnostic testing.

von Willebrand disorder diagnosis was confirmed in 16.1% (*n* = 10/62) of women with VWD who completed the LADY-X. No disease was present in 6.5% (*n* = 4/62) of the neonates, and no diagnostics was conducted in 77.4% (*n* = 48/62). There were 5 mothers who reported being worried about their child. Two of them knew their child had a bleeding disorder, 1 knew the child was unaffected, and 2 did not perform diagnostic testing. There were 7 mothers who delivered an affected child reported that not being worried during childbirth.

#### 3.3.2 Comparisons with the general population

Compared with the general Dutch population, HCs had more worries about their child (Figure 3D, Supplementary Tables 5 and 6). This was not the case in women with VWD.

## 4 Discussion

In this nationwide prospective cohort study, we prospectively assessed postpartum QoL and childbirth experience and satisfaction in Dutch HCs and women with VWD, for the first time. The QoL, as measured with the SF-36, showed significant improvement in HCs and women with VWD between week 1 and week 6 postpartum. Subjects were overall satisfied about their childbirth according to the MCSRS, significantly higher than the general population. Over 65% reported a positive childbirth experience on the LADY-X questionnaire. HCs, in contrast to women with VWD, had more worries about their neonate than the general population.

Compared with that in the general population, QoL was higher in HCs and women with VWD at 1 week postpartum, whereas at 6 weeks postpartum, the reported QoL was similar for all cohorts, with the exception for social functioning, role limitations due to emotions, and vitality, which were lower. These lower scores may be due to the resumption of daily tasks at week 6, where limitations became more apparent. At week 1, the new mother mostly focuses on her child and does not resume daily activities, while at 6 weeks postpartum, these activities are resumed, revealing the limitations. These limitation can be due to prolonged or excessive vaginal bleeding, which can also cause lower QoL at 6 weeks postpartum. Until cessation of vaginal bleeding, our participants received tranexamic acid orally to decrease the bleeding. However, in the PRIDES study, data on prolonged vaginal bleeding were not collected, but only when participants had late PPH needing medical attention; so, firm conclusions regarding the effect of prolonged bleeding at home on the QoL in week 6 cannot be drawn.

In our study, the rate of CSs was lower than that of the general population. CSs can result in more pain and reduces mobility. This may be an explanation as to why QoL compares favorably at week 1 in the bleeding disorder cohort. Both HCs and women with VWD had less pain at week 1. Over the years, the rate of analgesic effect during childbirth has increased, mostly by an epidural drug [21]. However, the effect of epidural drugs worn off at week 1 postpartum and did not explain the higher scores in our study.

Greater awareness of the potential for increased vaginal blood loss postpartum, along with longstanding and familiar heavy menstrual bleeding, potentially causes symptom normalization, whereby HCs and women with VWD may perceive their QoL as unaffected. This may partly explain the higher QoL reported 1 week after childbirth. At 6 weeks postpartum, this bleeding may result in postpartum iron deficiency anemia, revealing a slower recovery in HCs and women with VWD [22]. Prolonged and more than average vaginal bleeding past 24 hours, which was not measured in this study, may have contributed [23,24]. Late PPH with the need for medical attention occurred in only 7 women within the PRIDES cohort. We advise paying attention to postpartum physical and psychological well-being of HCs and women with VWD through follow-up visits and raising awareness for abnormal vaginal bleeding during this period. The Edinburgh Postnatal Depression Scale could be used to monitor postpartum depression [25].

Excessive bleeding and protocol based treatment of bleeding result in more medical interventions, possibly decreasing the mothers sense of control and possible lowering childbirth satisfaction. This has been reported by HCs [26]. Furthermore, hemophilia carriership and having VWD results in quicker conversions toward a CS and less assisted deliveries. Surprisingly, we observed overall high childbirth satisfaction, which was significantly higher than that in the general Dutch population. This may partly be due to improved obstetric care over time, particularly with the introduction of the new Integrated Maternity Care Standard in 2016 [[26], [27], [28], [29]]. Furthermore, negative childbirth experiences in the general Dutch population seem to have increased over the years, which is contrary to our results [9]. Personalized management in the form of a multidisciplinary birth plan and frequent consultations for HCs and women with VWD in hemophilia treatment centers may have contributed to higher satisfaction on the MCSRS [24].

HCs reported a worse childbirth experience regarding their worries about their child. Since their offspring can potentially be affected with hemophilia this finding is not surprising. Childbirth, especially instrumental deliveries, increases the risk of bleeding in these neonates, the worst being intracranial hemorrhage. HCs have previously reported to feel insecure about their delivery due to the increased bleeding risk for their child and the distance to the hemophilia treatment center. Additionally, fear of lack of knowledge of hemophilia by their health care professional causes insecurity [30]. Recorded prenatal diagnostic rate was low in our study, since we did not have informed consent to use the neonates medical record. Therefore, only if the diagnosis was reported in the mothers’ file, we could record it in our study. In our clinic, prenatal diagnostics is offered to all HCs and women with VWD with little or no response to desmopressin. If prenatal diagnostics was performed, a previous qualitative study with Dutch HCs revealed that most carriers refrained due to religious reasons, the risk for miscarriage, or impractical reasons (such as the hemophilia treatment center being far away). Since it is the same study population, we can assume that the same reasons are the same for our study. However, the limited data on prenatal diagnostics means we cannot use this as an explanation as to why HCs have more worries during childbirth. In women with VWD, childbirth experience was overall adequate and did not significantly differ from the general population. Time until the first contact with their child was not impacted, indicating that medical interventions or excessive bleeding had no influence. PPH has not been taken into account in this study but is separately collected as part of the PRIDES study. In previous research, PPH of ≥1500 mL did not significantly influence the outcomes of the SF-36 in a general population [31]. Severe PPH of ≥2000 mL has been proven to significantly increase the risk for PTSD in women without a bleeding disorder [5]. The influence of PPH on childbirth experience in women with inherited bleeding disorders needs to be further investigated.

### 4.1 Strengths and limitations

To our knowledge, this is the first study to assess postpartum QoL and childbirth satisfaction in women with VWD. Comparisons with the general population provide insights into health care areas for improvement, such as possibly slower recovery in the first 6 weeks after birth. A strength of the study is the relatively large cohort, allowing for more robust conclusions. The possibility that only women with good QoL and childbirth satisfaction choose to complete the surveys may have caused information bias. Still, a response rate of 70% is relatively high for survey studies, especially when taking the intense postpartum period into account. The nature of this study introduced limitations in comparing the current data with those of the historical cohorts. Variables that could influence childbirth satisfaction and QoL collected in the current study, such as parity, were not always available in the historic cohorts, which made correction for this in the analysis impossible. Another limitation is the focus on women treated at hemophilia centers, which may limit generalizability. However, women requiring clotting factor supplementation had to deliver in a hemophilia treatment center, allowing for easy inclusion in the study. Furthermore, ∼60% of the total PRIDES cohort completed one of the PROMs, introducing selection bias. Perhaps women with severe bleeding did not complete the questionnaire. The rate of women with severe postpartum bleeding who did not complete the PROMs were 52% in women with VWD and 28% in HCs. It was not possible to contact the subjects as to why they did not complete the questionnaires. Some PRIDES participants were retrospectively included during the COVID-19 period, and we chose not to let them complete the PROMs retrospectively to avoid a significant recall bias. Still, the response bias of those without severe PPH may have introduced an overestimation of the QoL and childbirth satisfaction. Additionally, using historic data for comparison reduces validity. Lastly, the SF-36 asks about a period of 4 weeks prior to moment of questionnaire completion. This may have led to an overestimation of QoL 1 week postpartum.

## 5 Conclusion

HCs and women with VWD experience good QoL and childbirth satisfaction in the postpartum period. QoL was decreased between week 1 and week 6 compared with that of the general population. A possible role of excessive postpartum bleeding needs to be investigated further. Childbirth satisfaction was significantly higher in HCs and women with VWD than that in the general population, possibly due to better management of expectations in women with a bleeding disorder. Future studies should focus on the role of postpartum bleeding on patient-reported outcome in HCs and women with VWD. We advise to monitor postpartum bleeding up to 12 weeks postpartum by follow-up consultations and pragmatically start iron supplementation in case of suspicion for iron deficiency anemia.
