# Supplementary material for: Postpartum well-being in hemophilia carriers and women with von Willebrand disease: insights from patient-reported outcome measures
Source: Res Pract Thromb Haemost. 2026 Mar 24;10(3):103418. doi: 10.1016/j.rpth.2026.103418 (PMC13098571; doi:10.1016/j.rpth.2026.103418)
Supplement: Supplementary Material [file mmc1.docx]

**Supplementary Table S1**. Difference in SF-36 outcomes between hemophilia carriers and the general population.

|  | SF-36 domain | Hemophilia carriers (n=85) | General population (n=141) | P-value |
| --- | --- | --- | --- | --- |
| Week 1 | General health | 80.9 | 75.2 | <0.001*** |
|  | Pain | 55.8 | 40.9 | <0.001*** |
|  | Social functioning | 66.3 | 57.2 | <0.001*** |
|  | Role limitations (Emotions) | 74.0 | 67.0 | 0.10 |
|  | Role limitations (Physical) | 40.0 | 22.0 | <0.001*** |
|  | Energy | 57.1 | 51.7 | <0.001*** |
|  | Emotional wellbeing | 80.0 | 79.0 | 0.41 |
|  | Physical functioning | 56.0 | 48.8 | 0.01* |
| Week 6 | General health | 78.3 | 78.0 | 0.53 |
|  | Pain | 71.1 | 78.2 | 0.63 |
|  | Social functioning | 71.3 | 86.2 | 0.002** |
|  | Role limitations (Emotions) | 75.7 | 82.7 | 0.16 |
|  | Role limitations (Physical) | 55.9 | 73.8 | 0.001** |
|  | Energy | 52.4 | 68.2 | <0.001*** |
|  | Emotional wellbeing | 79.2 | 86.3 | 0.06 |
|  | Physical functioning | 79.3 | 85.4 | 0.53 |

**Legend:** difference in mean quality of life score per SF-36 domain between hemophilia carriers and the general population. A one sample T-test was utilized. P-values <0.05 are considered as statistically significant. *n* number
Significance levels: * <0.05 ** <0.01 *** <0.001

**Supplementary Table S2**. Difference in SF-36 outcomes between women with VWD and the general population.

|  | SF-36 domain | Women with VWD (n=81) | General population (n=141) | P-value |
| --- | --- | --- | --- | --- |
| Week 1 | General health | 73.2 | 75.2 | 0.36 |
|  | Pain | 52.2 | 40.9 | <0.001*** |
|  | Social functioning | 62.9 | 57.2 | 0.08 |
|  | Role limitations (Emotions) | 63.5 | 67.0 | 0.48 |
|  | Role limitations (Physical) | 31.5 | 22.0 | 0.03* |
|  | Vitality | 49.9 | 51.7 | 0.45 |
|  | Emotional wellbeing | 76.4 | 79.0 | 0.13 |
|  | Physical functioning | 56.4 | 48.8 | 0.03* |
| Week 6 | General health | 70.7 | 78.0 | 0.02* |
|  | Pain | 71.1 | 78.2 | 0.03* |
|  | Social functioning | 67.7 | 86.2 | <0.001*** |
|  | Role limitations (Emotions) | 64.4 | 82.7 | <0.001*** |
|  | Role limitations (Physical) | 55.3 | 73.8 | <0.001*** |
|  | Vitality | 51.2 | 68.2 | <0.001*** |
|  | Emotional wellbeing | 78.1 | 86.3 | <0.001*** |
|  | Physical functioning | 80.0 | 85.4 | 0.05 |

**Legend:** difference in mean quality of life score per SF-36 domain between women with VWD and the general population. A one sample T-test was utilized. P-values <0.05 are considered as statistically significant. *n* number
Significance levels: * <0.05 ** <0.01 *** <0.001

**Supplementary Table S3**. Comparisons of the MCSRS between hemophilia carriers and the general population

| Mackeys Childbirth Satisfaction Rate Scale domains | Hemophilia carriers (n=70) n (%) | General population (n=611) n (%) | P-value |
| --- | --- | --- | --- |
| General | 70 (100) | 289 (47.3) | <0.001*** |
| Baby | 67 (95.7) | 232 (38.0) | <0.001*** |
| Self | 70 (100) | 426 (69.7) | <0.001*** |
| Physician | 62 (88.6) | 379 (61.9) | <0.001*** |
| Nurse | 70 (100) | 456 (74.6) | <0.001*** |
| Partner | 70 (100) | 456 (74.6) | <0.001*** |

**Legend:** Percentage ‘very satisfied’ and ‘satisfied’ hemophilia carriers and postpartum women from the general population on the Mackey Childbirth Satisfaction Rate Scale. Statistical difference assessed by a one sample test for proportions. P-values <0.05 are considered statistically significant. *n* number.
Significance levels: * <0.05 ** <0.01 *** <0.001

**Supplementary Table S4.** Comparisons of the frequency ‘satisfied’ and ‘very satisfied’ from the Mackey Childbirth Satisfaction Rate Scale between the cohort and the general population.

| Mackeys Childbirth Satisfaction Rate Scale domain | Women with VWD (n=64)  n (%) | General population (n=611)  n (%) | P-value |
| --- | --- | --- | --- |
| General | 64 (100) | 289 (47.3) | <0.001*** |
| Baby | 57 (89.1) | 232 (38.0) | <0.001*** |
| Self | 64 (100) | 426 (69.7) | <0.001*** |
| Physician | 58 (90.6) | 379 (61.9) | <0.001*** |
| Nurse | 61 (95.3) | 456 (74.6) | <0.001*** |
| Partner | 64 (100) | 456 (74.6) | <0.001*** |

**Legend:** Percentage ‘very satisfied’ and ‘satisfied’ hemophilia carriers and postpartum women from the general population on the Mackey Childbirth Satisfaction Rate Scale. Statistical difference assessed by a one sample test for proportions. P-values <0.05 are considered statistically significant. *n* number.
Significance levels: * <0.05 ** <0.01 *** <0.001

**Supplementary Table S5.** Frequency table of the Labor and Delivery Index outcomes of hemophilia carriers.

| N=59 | Very well  n (%) | Adequate  n (%) | Inadequate  n (%) |
| --- | --- | --- | --- |
| Presence of expert healthcare | 50 (84.7) | 9 (15.3) | 0 (0) |
| Information received | 43 (72.9) | 15 (25.4) | 1 (1.7) |
| Taking wishes seriously | 45 (76.3) | 12 (20.3) | 2 (3.4) |
| Emotional support | 48 (81.4) | 9 (15.3) | 2 (3.4) |
| Feeling of security | 43 (72.9) | 13 (22.0) | 1 (1.7) |
| Worries about child† | 23 (41.1) | 27 (48.2) | 6 (10.7) |
| Time until first contact with child | 53 (89.8) | 3 (5.1) | 3 (5.1) |

**Legend:** frequency table of the LADY-X domains. *n* number

**Supplementary Table S6.** Frequency table of the Labor and Delivery Index outcomes of women with VWD.

| N=62 | Very well  n (%) | Adequate  n (%) | Inadequate  n (%) |
| --- | --- | --- | --- |
| Presence of expert healthcare | 48 (77.4) | 13 (21.0) | 1 (1.6) |
| Information received | 42 (67.8) | 18 (29.0) | 2 (3.2) |
| Taking wishes seriously | 52 (83.8) | 8 (12.9) | 2 (3.2) |
| Emotional support | 49 (79.0) | 13 (21.0) | 0 (0) |
| Feeling of security | 48 (77.4) | 12 (19.4) | 2 (3.2) |
| Worries about child† | 42 (67.8) | 15 (24.2) | 5 (8.1) |
| Time until first contact with child | 58 (93.2) | 3 (4.8) | 1 (1.6) |

**Legend:** frequency table of the LADY-X domains. *n* number

**Supplementary Table S7.** Comparisons of the frequency ‘very well’ between hemophilia carriers and the general population.

|  | Hemophilia carriers (n=59)  n (%) | General population (n=295)  n (%) | P-value |
| --- | --- | --- | --- |
| Presence of expert healthcare | 49 (83.1) | 254 (86.1) | 0.86 |
| Information received | 42 (71.2) | 217 (73.6) | 1 |
| Taking wishes seriously | 43 (72.9) | 221 (74.9) | 1 |
| Emotional support | 46 (80.0) | 239 (81.0) | 1 |
| Feeling of security | 43 (72.9) | 226 (76.6) | 1 |
| Worries about child | 22 (37.3) | 213 (72.2) | <0.001*** |
| Time until first contact with child | 48 (81.4) | 273 (92.5) | 0.91 |

**Legend:** percentage of hemophilia carriers and women from the general population that reported ‘very well’ on the LADY-X domains. Analysis done by a chi-square test. P-value <0.05 is considered statistically significant. Significance levels: * <0.05 ** <0.01 *** <0.001. Abbreviations: *n* number

**Supplementary Table S8.** LADY-X outcomes compared between the cohort and the general population

| Labor and Delivery Index domain | Women with VWD (n=62)  n (%) | General population (n=295)  n (%) | P-value |
| --- | --- | --- | --- |
| Presence of expert healthcare | 48 (77.4) | 254 (86.1) | 0.06 |
| Information received | 42 (67.8) | 217 (73.6) | 0.27 |
| Taking wishes seriously | 52 (83.8) | 221 (74.9) | 0.13 |
| Emotional support | 49 (79.0) | 239 (81.0) | 0.59 |
| Feeling of security | 48 (77.4) | 226 (76.6) | 0.93 |
| Worries about child | 42 (67.8) | 213 (72.2) | 0.20 |
| Time until first contact with child | 58 (93.2) | 273 (92.5) | 0.98 |

**Legend:** percentage of women with VWD and women from the general population that reported ‘very well’ on the LADY-X domains. Analysis done by a chi-square test. Significance levels: * <0.05 ** <0.01 *** <0.001. *N* number
